# Integration of pulp and paper technology with bioethanol production

**DOI:** 10.1186/1754-6834-6-13

**Published:** 2013-01-28

**Authors:** Richard B Phillips, Hasan Jameel, Hou Min Chang

**Affiliations:** 1 Department of Forest Biomaterials, North Carolina State University, Box 8005, Raleigh, NC, 27695-8005, USA

**Keywords:** Biorefinery, Bioethanol, Co-located biorefinery, Enzyme hydrolysis, Greenfield biorefinery, Repurposed Kraft Mill

## Abstract

**Background:**

Despite decades of work and billions of dollars of investments in laboratory and pilot plant projects, commercial production of cellulosic ethanol is only now beginning to emerge. Because of: (1)high technical risk coupled with; (2) high capital investment cost relative to ethanol product value, investors have not been able to justify moving forward with large scale projects on woody biomass.

**Results:**

Both issues have been addressed by targeting pulp and paper industry processes for application in bioethanol production, in Greenfield, Repurpose and Co-Location scenarios. Processes commercially proven in hundreds of mills for many decades have been tailored to the recalcitrance of the biomass available. Economically feasible cellulosic bioethanol can be produced in Greenfield application with hardwoods, but not softwoods, using kraft mill equipment. Both types of wood species can profitably produce ethanol when kraft mill or newsprint assets are Repurposed to a biorefinery. A third situation which can generate high financial returns is where excess kraft pulp is available at a mill which has no excess drying capacity. Each scenario is supported by laboratory simulation, engineering and financial analysis. While pretreatment is critical to providing access of the biomass to enzymes, capital investment per unit of ethanol produced can be attractive, even if ethanol yield is modest.

**Conclusions:**

Three guiding principles result in attractive economics: (1) re-use existing assets to the maximum extent; (2) keep the process as simple as possible; (3) match the recalcitrance of the biomass with the severity of the pretreatment.

## Background

The literature (e.g. [1,2])documents more than 30 individual approaches to pretreatment of biomass to improve the efficiency of enzyme hydrolysis. The goals of pretreatment are:

1. Provide enzymes access to the carbohydrate fraction of biomass to maximize production of fermentable sugars;

2. Accomplish the first goal with minimum:

A. Loss of potential fermentable sugars;

B. Enzyme application;

C. Water carryover to fermentation and distillation;

D. Capital investment and operating cost.

E. Inhibitor formation (which is more problematic in acid, but less so in alkaline pretreatment processes where pulp is well-washed before entering enzyme hydrolysis and fermentation).

To date all public domain science and technical development have failed to achieve these goals on woody biomass, the most abundant and practical basis for a bioethanol industry. More difficult still are softwood species compared to hardwoods.

**Figure 1:**
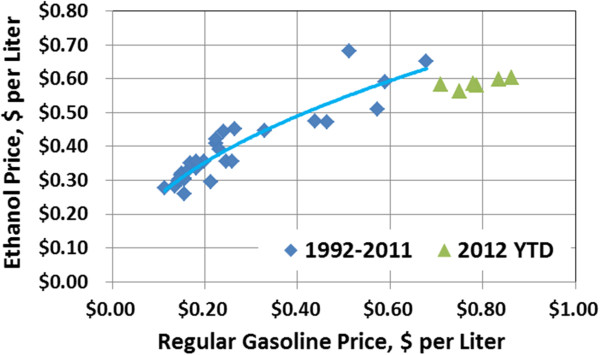
**Ethanol and gasoline volumetric selling price (1992 – June, 2012, YTD), Rack Prices, Omaha, NE**[3].

Pretreatment is an important – but by no means only step required for commercial success (defined here as technologies that can achieve economically financeable bioethanol projects not requiring subsidies, government grants, or other marketplace distortions once demonstrated). Economically financeable means the investors should expect a 12% Internal Rate of Return (IRR) on their capital with a market price of ethanol on par with gasoline on a fuel value equivalent basis (a target of $0.65 per Liter [$2.46 per gallon] would equate to gasoline at $0.98 per liter [$3.80 per gallon]). The most comprehensive database comparing ethanol and gasoline prices has been maintained by the Nebraska Energy Office [3], which reports monthly rack prices for each fuel in Omaha. Figure 1 indicates that ethanol sold for a premium over gasoline on a volumetric basis for many years, but the loss of the blender credit and reaching the 10% “blend wall” in 2012 has led to ethanol selling in recent months at the expected discount to regular gasoline. Investors would likely require any new process in the United States achieve the 12% with a minimum ethanol revenue (MER) around $0.60 - $0.65 per liter. Table 1 summarizes assumptions used in deriving MER for a number of scenarios evaluated in this study.

**Table 1:** Summary of cases studied at North Carolina State University for industrial application of pulp mill technology to Biorefining

**Main financial assumptions**	
**Item**	**Rule**
**General**	Pro forma investment analysis based on either: (1) Greenfield project that starts with land acquisition in 2010, and includes all investments as negative cash flows; or (2) Repurposed Mill based on owner transferring assets at no cost to ethanol facility (justified by equality of asset scrap value versus site closure and environmental remediation costs). Production begins June 1, 2012 in all cases.
**Capital Investment**	Case specific investments were developed for $2012, the startup year. Capital spending in each case was 30% in 2010, 50% in 2011, and 20% in 2012.
**Depreciation**	Based on 7-year MACRS depreciation.
**Project Life**	15 years
**Terminal Value**	5 x EBITDA of the terminal year (2027)
**Replacement Asset Value (RAV)**	Benchmark was calculated on the basis of the estimated cost of reproducing the assets each future year. Calculated by assuming 3% annual increase in the installation cost. Repurposed options include RAV on the same basis including only the assets that are reused.
**Reinvestment Capital**	1% of RAV reinvested as capital each year in order to maintain existing capability.
**Maintenance Expense**	2% of RAV included annually to account for maintenance labor and materials.
**Other Fixed Costs**	3% of annual sales
**Overhead Costs**	3% of annual sales
**Labor Costs**	Based on technology-specific salaried, operating and administrative staff.
**Tax**	35% overall tax rate on profit, with tax losses accumulated and carried forward to offset profits made in future years.
**Working Capital**	10% of all Direct Costs + pre-subsidy (if any) ethanol revenue.
**Net Present Value**	All Free Cash Flows (Cash Flows less new fixed capital and change in working capital) are discounted at 12% to the startup year. The ethanol revenue required to achieve Zero NPV (12% Internal Rate of Return) are used for Minimum Ethanol Revenue (MER).
**Biomass Cost**	Case specific costs for 450,000 BDt deliveries were taken from the plantation economics sub-study (Gonzalez [].
**Pretreatment Yield**	Pretreatment-specific yields were input from laboratory studies referenced earlier.
**Post Treatment Yield**	Post treatment yields (including mechanical refining and oxygen delignification) were input from the laboratory studies referenced earlier.
**Enzyme Hydrolysis Yield**	Enzyme Hydrolysis yield of monomeric sugars was input from laboratory studies. A dose of 5 FPU per gram of substrate was used in all cases. Cost of enzyme assumed to be $1.00 per Kg of Enzyme Product.
**Fermentation Yield**	80% fermentation of 5-carbon sugars and 95% fermentation of 6-carbon sugars.
**Raw Material Pricing**	Raw Material pricing and indices input from chemical marketing and forecasts
**Improvement in Productivity / Inflation**	Each component of cost was individually assigned an “annual productivity factor” based on Best Professional Judgment, and an annual escalation factor of the unit costs. These refinements have little impact on final outcome. Ethanol Revenue was calculated as above as Minimum Ethanol Revenue (MER), which was escalated at 3% per year, assuming the same rate generally considered for gasoline and crude oil.

Our efforts at North Carolina State University [4-19] take advantage of the technology developed over for more than one hundred years in the kraft and newsprint paper industries. While ethanol production has been practiced at sulfite pulp mills for more than one hundred years, we are not aware of commercial activities outside that increasingly diminished population of pulp mills. We have published a number of technical / economic studies of repurposing unprofitable mills to the production of ethanol, with a focus on combinations of biomass and simpler subsets of processes that can achieve technical and financial feasibility. Simplicity – meaning the least number of unit operations that must be capitalized and operated - is the main way to break through the capital investment barrier that stands in the way of many otherwise exciting approaches to pretreatment. We look for combinations of biomass, capital, ethanol yield and enzyme doses that can achieve the targeted Minimum Ethanol Revenue.

## Results and discussion

### Pulp and Paper Mill repurposing

**Figure 2:**
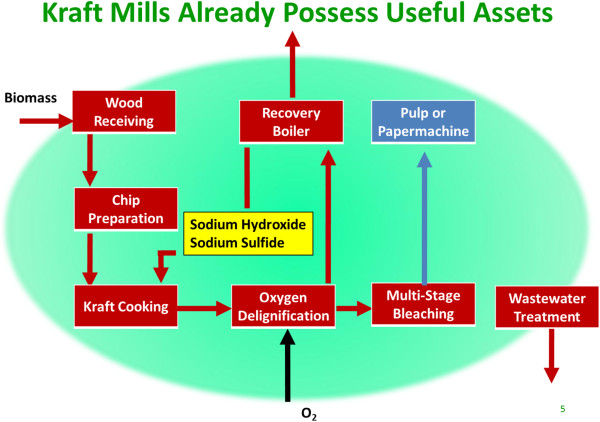
**Basic kraft pulp and paper mill process flow diagram.** Brown units are candidates for Repurposed mill application.

Many of the Unit Operations – wood handling, digesting, mechanical refining, evaporation, combustion - proposed for any pretreatment exist already in Kraft Pulp Mills (Figure 2), using chemistry, technology, and process equipment proven for more than a hundred years. When using acid, this approach has less value, but there are advantages to alkaline pretreatments that have been documented in the literature, not the least of which is avoidance of enzyme hydrolysis and fermentation inhibitors [20]. Mildly acidic (greater than pH 3) processes, such as autohydrolysis with hardwood, can also be accomplished in mild steel equipment typical of pulp and paper mills. Often understated is the value of having a complete chemical and energy recovery system built into the design of every kraft mill in the world. The real questions on this approach are: (A) carbohydrate preservation – generally not as good as other pretreatments; and (B) applicability to a greenfield or co-located installation as opposed to repurposing. Carbohydrates are lost in alkaline pretreatments by the well know alkaline “peeling” reaction that results in loss of hemicelluloses [21].

**Figure 3:**
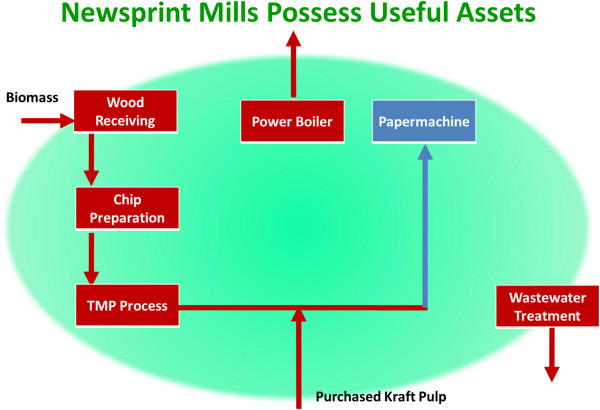
**Process flow diagram for typical newsprint mill.** To be a candidate for Repurpose to Biorefinery, the mill must have TMP or BCTMP capability. Most newsprint mills process softwood for mechanical pulps, while only hardwoods are amenable for Biorefinery purposes.

Newsprint mills (Figure 3) – over 30 of which have shut down over the past 5 years in Eastern Canada and Northeastern United States [22] – have fewer assets to redeploy, but our efforts have found that southern hardwoods and non-wood biomass can achieve relatively high yields – on the order of 250–300 liters per BDt of biomass – using multiple stages of autohydrolysis as pretreatment. No kraft pulp chemistry or chemical recovery boiler is required for this option.

**Figure 4:**
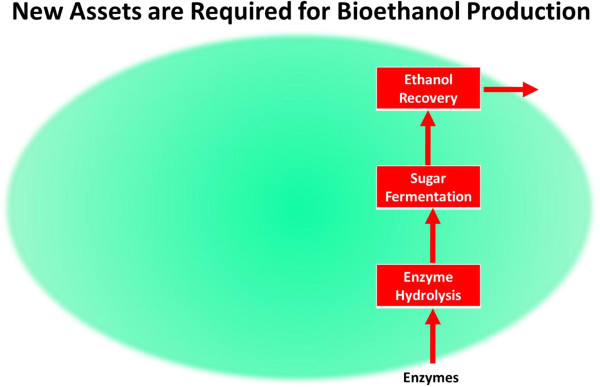
New process steps required to be added to kraft pulp or newsprint mills to achieve Biorefinery application.

Whether kraft or newsprint mills are the target, new equipment (Figure 4) is required to process fiber through enzyme hydrolysis, fermentation and ethanol recovery. In this regard, capital investment for the biorefinery portion of the plant can be maintained at levels on the order of a corn to ethanol plant. Many biochemical processes described in the literature exhibit low sugar solids which drives fermentation and distillation capital investment and energy costs to unattractive levels. Key to economics is the ability to process pulp fibers at 10-15% solids into enzyme hydrolysis to minimize capital and energy that are expended to accommodate water loads. We have developed several alternatives for doing this efficiently, and applied for U.S. patents on the inventions [18,19].

### Kraft mill repurposing

**Figure 5:**
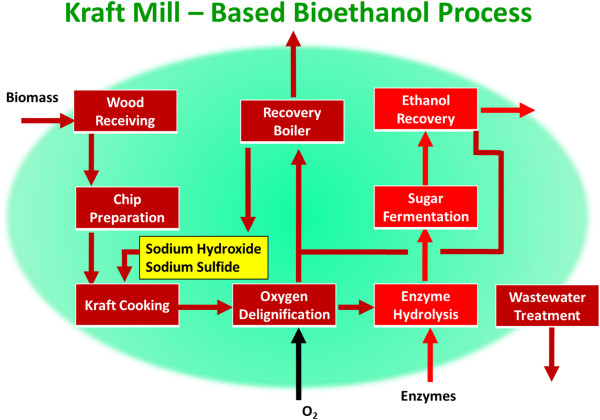
**Kraft Pulp Mill – based Biorefinery.** Brown units are typically found in kraft pulp mills which can be Repurposed to Biorefinery application, or must be built new in a Greenfield application.

Putting it all together in a kraft mill context is shown in Figure 5. For hardwoods, the severity of kraft pulping is not required to overcome recalcitrance of hardwood [19], and instead the less severe and less expensive Green Liquor approach (where only sodium sulfide and sodium carbonate are employed) should be considered. Whether kraft or green liquor pretreatment, both approaches are too capital – intensive to qualify for Greenfield projects, but both are quite interesting in the Repurpose context.

### Newsprint mill repurposing

**Figure 6:**
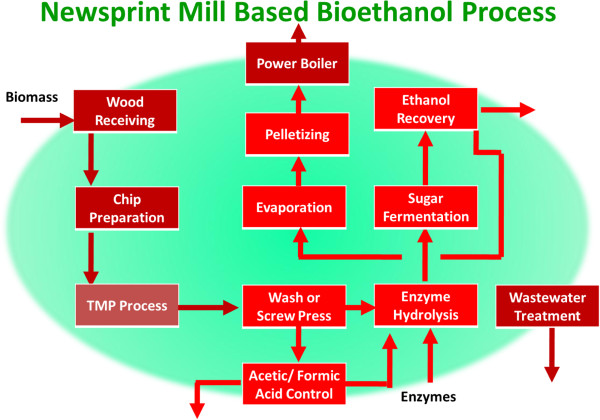
**Newsprint Mill – based Biorefinery.** Brown units are typically available in newsprint mills, or must be built in a Greenfield application.

For the abandoned newsprint application (Figure 6), autohydrolysis must be carried out with excellent control of temperature – no greater than 180°C. At this temperature, acetyl groups present in most hardwoods at 2-3% concentration are hydrolyzed from hemicellulose and go into solution as acetic acid [21]. A conventional TMP or BCTMP cooking vessel is contemplated, discharging through a high energy refiner. Refined pulp is typically conveyed to a second stage of refining, where we have found significant improvements in enzyme hydrolysis efficiency. We propose to cool and wash the pulp fibers, using internal process streams, and recycle the wash press filtrate through ion exchange resins to control acetic acid concentration. Small amounts of acetic acid can be tolerated in enzyme hydrolysis and fermentation; the control scheme described above is adequate to meet normal fermentation system requirements. Other potential inhibitors are controlled by the steam stripping that occurs during digester blow, similar to the steps taken in Dilute Acid treatment [23].

Without inorganic chemical use, the recovery requirement – and capital investment - of a kraft mill is not necessary in autohydrolysis. Following fermentation, Beer Column Bottoms are captured and clarified through gravity sedimentation. Ultimately, lignin is recovered by evaporation and spray drying. We visualize processing lignin and unfermented sugars to near dryness for efficient combustion in a power boiler. Preliminary balances suggest that the residue can provide 100% of process steam, and if processed through a turbine-generator, allows the mill to be self-sufficient in power. Alternatively, especially if low cost natural gas is available, the residue can be converted to a high energy density pellet for a higher valued product that can be co-fired with coal in a commercial power generating facility.

Autohydrolysis is very attractive economically on hardwoods or non-wood plants, but also can approach the target Minimum Ethanol Revenue for a Greenfield project. Autohydrolysis with softwoods thus far does not appear attractive today without some breakthrough.

## Post treatments

**Figure 7:**
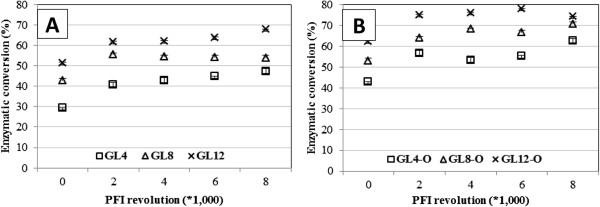
**Enzyme hydrolysis improvement through the use of mechanical refining alone (7a) and refining + oxygen delignification (7b).** Enzyme hydrolysis efficiency based on carbohydrate content in pretreated substrate (77%). From Koo [13]. GL = Green Liquor Pretreatment”. GL-4 = “Green Liquor Pretreatment with 4% Total Titratable Alkali”. GL-4-O = “Green Liquor Pretreatment with 4% Total Titratable Alkali + Oxygen Delignification”.

All pretreatments are followed by a post treatment of fibers with conventional pulp and paper mill mechanical refining (Figure 7a) and oxygen delignification (Figure 7b). Although refining could consume on the order of 200 KWH per BDt of pulp, the reduction in enzyme dose is a good economic tradeoff; if an existing mill did not have oxygen delignification originally, we find it is financially justified to build a new system.

Mechanical refining [13] is a practical way to improve access of enzyme to fibers without loss of carbohydrates in pretreatment. Steam Explosion accomplishes improved accessibility through pretreatment, we do it as post treatment. Xue [11] and Phillips [18] observed that the key to rapid enzyme hydrolysis at high solids is to achieve fiber network destructuring early in the process, so that the mobility of fibers and enzymes is increased. Where an alkaline recovery system is in place, further delignification with oxygen after pretreatment has been found to increase the level of enzyme hydrolysis efficiency by selectively removing large amounts of lignin without losing carbohydrates.

### The challenge of softwoods as feedstock

**Figure 8:**
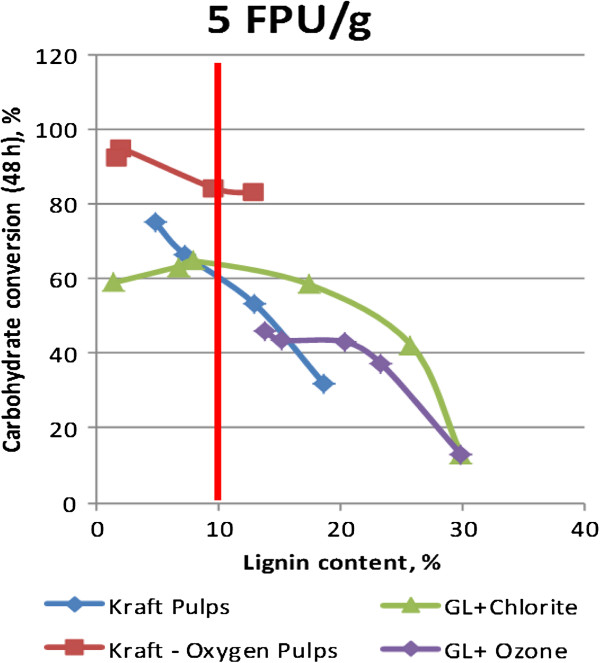
**Compilation of results from Yu and Wu [to be published].** Pulps at same lignin content behave marked different in enzyme hydrolysis, dependent on the chemistry of the pretreatment. Kraft – Oxygen pulps respond most efficiently.

Hardwoods can be effectively processed with Green Liquor or Autohydrolysis, without significant removal of lignin, but softwoods appear very recalcitrant until lignin content has been reduced below 10% (Figure 8). In addition, exactly how the lignin is removed is important. Ozone and chlorine dioxide are relatively inefficient compared to kraft pulping, but oxygen delignification appears to provide a boost at every lignin level. The enzyme hydrolysis efficiency appears to parallel independent measurements of surface area. It is well known that enzymes bind preferentially to lignin [15]; these results point to importance of the chemical structure of residual lignin as a factor.

At least for the southeastern United States, producing bioethanol from loblolly pine is the ultimate prize. With wood cost in the $60-70 per BDt range, with forecast of oversupply for years to come, some level of inefficiency can be tolerated. So rather than focusing on the difficult challenge of achieving 300 + liters per BDt with pine, we have explored opportunities accepting simpler processes with lower ethanol yields from kraft pulping as pretreatment.

**Figure 9:**
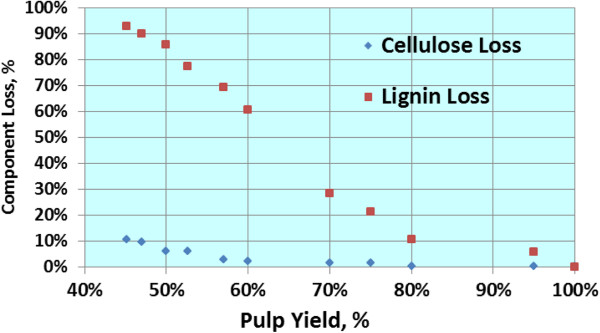
**Retention of glucan and lignin as a function of softwood pulp yield.** Even with 90% lignin removal, Glucan retention is >90%. Derived from Rydholm [21].

**Figure 10:**
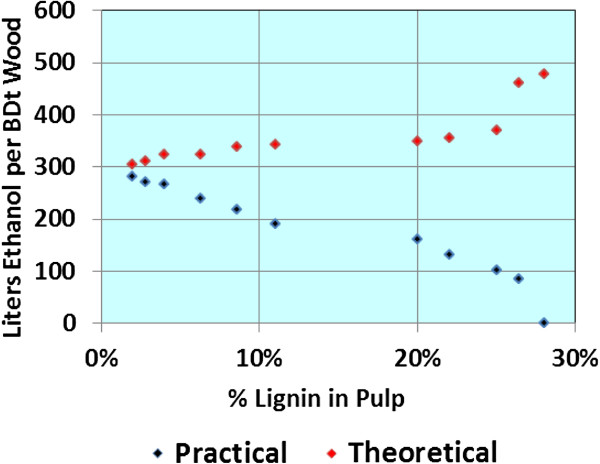
**Theoretical yields are calculated on the basis of 100% conversion of Glucan, Mannan (and other 6-Carbon sugars), and Xylans (and other 5-Carbon sugars) in enzymatic hydrolysis, and conversion of the sugars in ethanolgenic fermentation at 100% fermention efficiency.** Practical yields are estimated based on application of 5 FPU enzyme (per gram of substrate), laboratory conversion efficiency, and 95% and 80% conversion of C-6 and C-5 sugars, respectively.

Kraft pulping is known to be selective for glucan retention (Figure 9, see, for example, Rydholm [21]), but less so for either mannans or xylans. Converting kraft pulps through enzyme hydrolysis to sugars requires finding the proper balance between high lignin content (and high carbohydrate content) and high enzyme hydrolysis efficiency (lower carbohydrate content but high conversion). Practical ethanol yield approaches theoretical ethanol yield at the low lignin levels, albeit, below 300 liters per BDt (Figure 10).

However, to achieve those yields with economically affordable enzyme doses, we find that oxygen delignification, as well as mechanical refining are helpful. The effect of oxygen delignification – a proven technology – is profoundly important in achieving readily hydrolysable fibers while preserving carbohydrate yield.

Complete Repurposing a kraft mill is quite feasible, but requires the mill to be closed to do so. Closures in North America, Western and Northern Europe will continue as demand for paper declines, but the plant conversion to ethanol must occur quickly before the assets deteriorate. We have looked at this with financial partners on several occasions and observed how rapidly unpreserved assets degrade.

### Co-location

**Figure 11:**
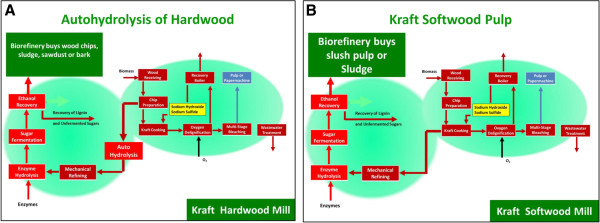
**Co-Location options.** (**A**) Autohydrolysis of hardwood; (**B**) kraft pulping to <10% lignin content.

Other “mini-repurposing opportunity exists with kraft mills that produce pulp in excess of what can be converted to paper or board. In particular, as industrial linerboard basis weights continue to be reduced, a number of mills can find 50–100 tpd of excess pulp available for bioethanol production. Small volumes would be produced, but represents a low capital start that might eventually encompass the entire mill. In addition these plants might process fibrous sludge and sawdust, upgrading their value past the point of boiler fuel. Figure 11 shows schemes for co-location with autohydrolysis (A) and kraft pulp (B).

### Green liquor pretreatment

Table 2 is typical output of our technical / economic simulations. These cases compare Green Liquor Pretreatment of mixed southern hardwoods on both a Greenfield and Repurpose basis. Generally we find little differences only in power plant performance between Greenfield and Repurpose mills: the major difference is in capital investment ($329 versus $80 Million, respectively. These differences translate to a difference in Minimum Ethanol Revenue of $0.83 versus $0.52 per Liter.

**Table 2:** Financial summary of green liquor pretreatment of hardwood

**Hardwood green liquor cases**				
	**2015 Projection**		**2015 Projection**	
	**Greenfield GL Mill**		**Repurpose GL Mill**	
	**Quantity**	**Cost per unit**	**Quantity**	**Cost per unit**
Mixed Southern Hardwood	454,545	$72.18	454,545	$72.18
Annual Ethanol Production, Liters	148,072,272		147,905,135	
Ethanol Yield, Liters per BDt	326		325	
CAPEX Total/per Annual Liter	329,494,169	$2.22	$79,955,469	$0.54
Total Biomass Cost per Liter		$0.24		$0.24
Total Enzyme Cost per Liter		$0.12		$0.12
Total Energy Credit/cost		$0.04		$0.02
Total Direct Cost per Liter		$0.33		$0.35
Total Indirect Cost per liter		$0.50		$0.18
Total Cash Cost per liter		$0.52		$0.45
Total Cost per liter		$0.83		$0.54
MER,$ per liter		$0.83		$0.52
IRR, 0025		12%		12%

No major differences in plant performance but investment cost in the Repurpose case permits Minimum Ethanol Revenue below the target of $0.60 per Liter.

Table 3 shows the comparable analysis of loblolly pine Green Liquor pretreatment. The low yield of ethanol from loblolly pine, coupled with high enzyme dose (2 x the level of hardwood) dooms this approach to softwood biorefinery.

**Table 3:** Financial summary of green liquor pretreatment of loblolly pine

**Green liquor loblolly pine**				
	**2015 Projection**		**2015 Projection**	
	**Greenfield GL Mill**		**Repurpose GL Mill**	
	**Quantity**	**Cost per unit**	**Quantity**	**Cost per unit**
Loblolly Pine	454,545	$69.28	454,545	$70.87
Annual Ethanol Production, Liters	123,894,322		125,534,368	
Ethanol Yield, Liters per BDt	273		276	
CAPEX Total/per Annual Liter	309,ʓ ,845	$2.50	$75,741,067	$0.60
Total Biomass Cost per Liter		$0.27		$0.28
Total Enzyme Cost per Liter		$0.16		$0.36
Total Energy Credit/Cost		$0.02		-0.05
Total Direct Cost per Liter		$0.44		$0.60
Total Indirect Cost per liter		$0.44		$0.22
Total Cash Cost per liter		$0.60		$0.71
Total Cost per liter		$0.88		$0.81
MER,$ per liter		$0.94		$0.78
IRR, 0025		12%		12%

Despite the investment cost difference, Repurposing an existing kraft mill to produce bioethanol from loblolly pine is not economical.

### Opportunities with hardwood

Autohydrolysis of hardwood is quite efficient as discussed earlier. Previous studies of autohydrolysis have not been encouraging, perhaps due to two areas which are corrected in our scheme. First, mechanical refining increases enzyme hydrolysis efficiency by as much as 25%, thus improving yield (though not to the level of Green Liquor). Second, control of furfural and acetic acid byproducts of prehydrolysis must be controlled in a cellulose- water system. By employing a screw press following autohydrolysis, and removing large quantities of pretreatment dissolved solids, including acetic and formic acid, we can separately and efficiency remove through membranes or ion exchange columns the chemicals which may interfere with fermentation. Furfural and hydroxymethyl furfural are steam stripped at 70% efficiency from autohydrolysis in the digester blow process, based on Reference [23].

Table 4 displays autohydrolysis on both a Greenfield basis, and as a retrofit in a Repurposed Newsprint Mill, shifted from the typical softwood to a hardwood feedstock. Very high yields of ethanol are achieved, which we attribute to the impact of mechanical refining on increasing enzyme accessibility. While the Flowsheet shows lignin and other fermentation residue recovered in a biomass boiler, pelletizing and capturing the higher market with a high value fuel for co-firing with coal improves the economics.

**Table 4:** Use of autohydrolysis of mixed Southern hardwoods to produce sugar-laden hydrolysate and autohydrolysis residue

**Comparison of autohydrolysis options**				
***Hardwood Pretreatments***				
	**Mixed Southern hardwood**		**Mixed Southern hardwood**	
	**2-Stage Autohydrolysis**		**Newsprint Repurpose**	
	**Quantity**	**Cost per unit**	**Quantity**	**Cost per unit**
Mixed Southern Hardwood	454,545	$72.18	454,545	$72.18
Annual Ethanol Production, Liters	141,771,687		122,880,735	
Ethanol Yield, Liters per BDt	312		270	
CAPEX Total/per Annual Liter	213,776,300	$1.50	$117,823,560	$0.96
Total Biomass Cost per Liter		$0.25		$0.29
Total Enzyme Cost per Liter		$0.11		$0.12
Total Energy Credit/Cost		$0.00		-0.01
Total Direct Cost per Liter		$0.37		$0.43
Total Indirect Cost per liter		$0.36		$0.33
Total Cash Cost per liter		$0.51		$0.61
Total Cost per liter		$0.73		$0.76
M. E. R,$ per liter		$0.71		$0.73
IRR, 0025		12%		12%

The residue is mechanically refined to improve accessibility to enzymes. The hydrolysate is treated with ion exchange resins to remove acetic acid, and then combined with the residue for enzymatic hydrolysis (see Figure 4).

The autohydrolysis approach studies here is distinctly different from that taken in recent years to convert kraft paper pulp mills to prehydrolysis kraft dissolving pulp mills. While both approaches employ water prehydrolysis to remove hemicellulose, we do not employ a pulping step. The dissolving pulp market is currently over-supplied by kraft mill conversions, while the ethanol market is not.

### Opportunities with softwood

Most researchers have found softwoods more recalcitrant to enzyme action than hardwoods, and even more so than nonwood biomass. Our results shown above with Green Liquor pretreatment illustrate the magnitude of the problem. Applying our principle of “match the recalcitrance of the biomass with the severity of the pretreatment” led us to conclude (as illustrated in Figure 10) kraft pulping might be the optimum pretreatment for softwoods. Typically, softwood requires 2- to 3 times the enzyme dose to achieve even a modest 70% sugar conversion, but kraft pulping prepares the pulp fibers very well for high conversions with low doses.

We analyzed two practical approaches to employing kraft pulping pretreatment: (1) Processing a small line of kraft pulp in an operating mill through oxygen delignification and mechanical refining prior to enzyme hydrolysis (“Co-Location”); (2) repurposing a kraft mill to ethanol production, but using kraft pretreatment instead of Green Liquor (“Repurposing”).

#### Processing sidestream of kraft pulp in an existing linerboard mill

**Figure 12:**
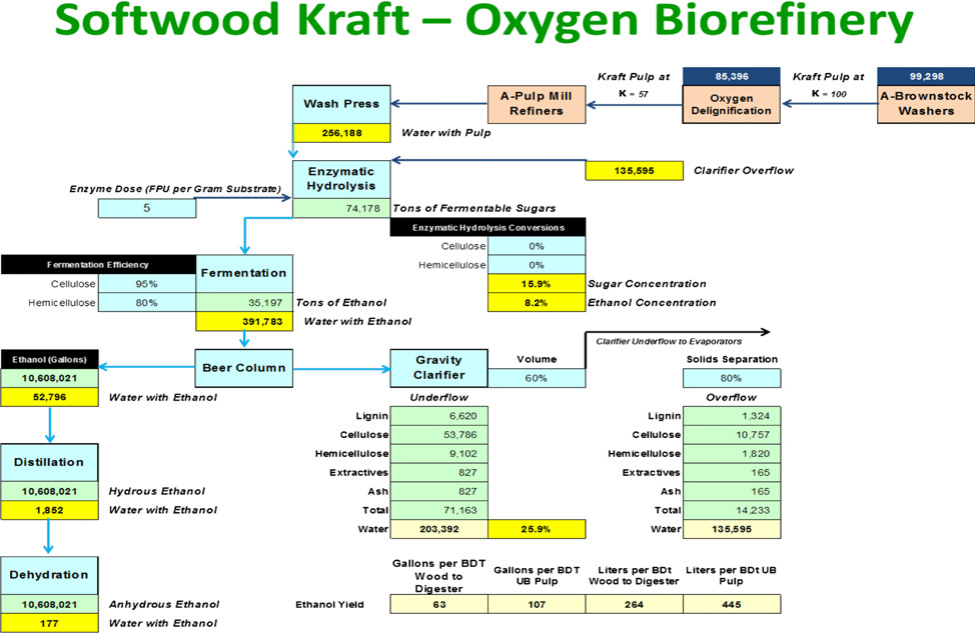
**Mass balance and process scheme for converting 100,000 short tons per year of linerboard pulp at 15% lignin (100 kappa number) into 264 liters per BDt of pulp of bioethanol.** Oxygen Delignification is not likely to be available in a linerboard operation, and the wash press illustrated would also be a new investment.

This option cannot be financially attractive if a mill is kraft fiber limited in a linerboard environment. However, lightweight linerboard production is gradually increasing at the expense of heavier weight grades. Customers can purchase 35% more area per ton of linerboard at 26 pounds per 1,000 Ft^2^, than the new normal of 36 pounds and 41% more than the old normal of 42 pounds. However at the linerboard mill, machines would have to speed up in order to keep annual output up at the same level achieved with heavier weight grades. Some mills have made the considerable investment to do so, while others may find the cost and complexity prohibitive. Figure 12 illustrates one mill study we completed. Yield of ethanol per BDt of pulp is quite attractive (445), but on a BDt of the original wood is 264 liters, consistent with Figure 10.

#### Repurposing a softwood Kraft pulp mill

Earlier, we saw that Green Liquor was inadequate to pretreat loblolly pine, even with oxygen delignification and mechanical refining post treatments. However, Figure 10 guides us towards low lignin content kraft pulping (to reduce enzyme dose) accepting that the yield of ethanol from mannans and xylans will be lower than achieved in Green Liquor pretreatment. However, one feature of a kraft mill that lends itself to making this concept work is the highly efficient chemical and energy capability of the kraft recovery system. Though ethanol yield is relatively low compared to hardwoods, both the “Excess Kraft Pulp” and the “Repurposed Kraft Pulp Mill” options achieve very attractive financial performance.

#### Future work

Repurposing is particularly attractive as a route to finance bioethanol production with low financial and technical risk. In some respects, all the technology we would employ in pretreatment is commercially proven for decades; the technology employed in fermentation and ethanol recovery is widely practiced. The steps in between need proof on concept at a larger scale, but are no more difficult than any other cellulosic ethanol alternative that has been proposed elsewhere. The challenges facing commercial implementation of the concepts discussed in this paper are: (1) funding to complete the demonstration of the enzyme hydrolysis efficiency at high solids loading; and (2) the availability of facilities that are partly or completely closing kraft pulp, or newsprint lines. Facility availability must be identified before the facility is closed so that asset preservation steps are undertaken.

A number of clear advantages of Repurposing in addition to capital investment include:

1. Reduced project cycle, since infrastructure and many assets are already in place.

2. Trained workforce in place, motivated for long term success with a product that has enduring demand.

3. A fiber supply structure in place.

4. Local and other government support likely to avoid economic hardships in a region.

## Conclusions

Both kraft pulp and newsprint mechanical pulping mills can be repurposed to bioethanol production, and produce attractive economics. Technical and equipment risk are minimal for Pretreatment, but commercial demonstration of enzyme hydrolysis at high solids, and fermentability of sugars is required. Ethanol yields in repurpose applications may not achieve levels with more complex pretreatments, but economically justified ethanol can be produced without subsidies. Each biomass type presents a different challenge to overcoming biomass recalcitrance: for example, autohydrolysis is the best economic solution for hardwoods, but kraft pulping is best-suited for softwood.

Based on observations presented, we find three guiding principles that result in attractive economics: (1) re-use existing assets to the maximum extent; (2) keep the process as simple as possible; (3) match the recalcitrance of the biomass with the severity of the pretreatment.

### Methodology

The Greenfield and Repurpose Mill concepts described above have all been simulated at the laboratory level over the past five years, with details and analytical approaches described elsewhere [2-17]. The goal of this report is to collect the results of engineering and financial modeling we have developed using the laboratory findings.

In all cases, we have a biomass model, a process simulation model, and a financial model.

### Biomass Model

The delivered cost of any biomass to a production facility is a result of the land cost, the planting and maintenance cost of the crop, the harvesting cost, and the transportation cost. For woody species, these costs are well known from many public sources. Each production facility will have a wide variation in costs, dependent on the local circumstances, most particularly the density of the biomass plantation and the distance from the plant. Gonzalez has provided detailed methodology for a wide variety of biomass types. The discussion below is based on a specific circumstance for loblolly pine and mixed southern hardwood, with delivered costs for 450,000 Bone Dry Metric Tonnes (BDt) of $70.87 per BDt and $72.18, respectively. An additional case of a Canadian Newsprint Mill was developed, with an assumed cost of $81.48 per BDt, a value provided by FisherSolve™, a well-regarded paper industry database [22].

### Process simulation

Each process was incorporated into standard pulp and paper industry mass and energy balances using winGEMS [24]. The simulation required input data from the laboratory simulations referenced earlier. Output from winGEMS included raw materials, chemicals, and energy. Most cases were energy-positive in that recovered lignin and unfermented carbohydrates were burned to produce steam. Steam was passed through a turbine-generator set, with the excess of process demands condensed to generate excess power for sale.

The equipment requirements, the flows, and estimated manning requirements were developed using factored engineering estimates from pulp and paper industry projects, and NREL 2011 corn stover biorefinery update [23] for biorefinery equipment.

### Financial simulation

Standard investment finance techniques were used, with the specific parameters displayed in Table 1. In brief, the capital investments were spread over a three year period prior to and including 2012, the year of plant startup. Maintenance and Other Mill Fixed Costs were estimated as a function of the Replacement Asset Value (the original investment escalated annually in cost at 3% to represent the total cost to rebuild the existing fixed assets). Operating costs were developed based on the winGEMS outputs and current chemical costs [22]. Enzyme cost was estimated from the scant public information [25] as follows: Novozymes has indicated enzyme costs for corn stover in 2012 at $0.50 per Gallon. Assuming best literature value of 4 FPU enzyme per gram of glucan, assuming CETEC II as the subject enzyme, and assuming a 50% improvement in cost going forward (both efficiency and cost of enzyme), we derived an estimate for our lab work of $0.14 enzyme cost per liter per FPU per gram of substrate, assuming enzyme product cost of $1.00 per Kilogram. Admittedly speculative, this estimate is the best we can do with limited access to real pricing.

Investments (depreciated using a 7-Year Modified Accelerated Depreciation schedule (MACRS) and costs were input to the financial model, and cash flows and free cash flows were calculated for 15 years. Rather than assuming a revenue price of ethanol, we used the financial model to back calculate the Minimum Ethanol Revenue (MER) required to yield a 12% Internal Rate of Return to the plant investment.

Thus, different alternatives can be compared on the basis of MER, with the best options reflecting the lowest MER.

## Competing interests

The authors declare that they have no competing interests.

## Authors’ contributions

RP performed the engineering and financial analysis reported. HJ supervised the laboratory experimentation supporting the analysis. HMC provided direction on the overall concept and enzyme hydrolysis optimization. All authors read and approved the final manuscript.
